# Impact of monovalent rotavirus vaccine on diarrhoea-associated post-neonatal infant mortality in rural communities in Malawi: a population-based birth cohort study

**DOI:** 10.1016/S2214-109X(18)30314-0

**Published:** 2018-08-10

**Authors:** Naor Bar-Zeev, Carina King, Tambosi Phiri, James Beard, Hazzie Mvula, Amelia C Crampin, Ellen Heinsbroek, Sonia Lewycka, Jacqueline E Tate, Umesh D Parashar, Anthony Costello, Charles Mwansambo, Robert S Heyderman, Neil French, Nigel A Cunliffe, VacSurv Consortium, Osamu Nakagomi, Jennifer R Verani, Cynthia G Whitney

**Affiliations:** a Centre for Global Vaccine Research, Institute of Infection and Global Health, University of Liverpool, Liverpool, UK; b Malawi-Liverpool-Wellcome Trust Clinical Research Programme, College of Medicine, University of Malawi, Chichiri, Blantyre, Malawi; c International Vaccine Access Center, Johns Hopkins Bloomberg School of Public Health, Baltimore, MD, USA; d Mai Mwana Project, Mchinji, Malawi; e Institute for Global Health, University College London, London, UK; f Division of Infection and Immunity, University College London, London, UK; g Parent and Child Health Initiative, Lilongwe, Malawi; h London School of Hygiene & Tropical Medicine, London, UK; i Malawi Epidemiology and Intervention Research Unit, formerly Karonga Prevention Study, Chilumba, Malawi; j US Centers for Disease Control and Prevention, Atlanta, GA, USA; k Child and Adolescent Health, World Health Organization, Geneva, Switzerland; l Ministry of Health, Lilongwe, Malawi

## Abstract

**Background:**

Rotavirus is a major contributor to child mortality. The effect of rotavirus vaccine on diarrhoea mortality has been estimated in middle-income but not low-income settings, where mortality is high and vaccine effectiveness in reducing admissions to hospital is lower. Empirical population-based mortality studies have not been done in any setting. Malawi introduced monovalent rotavirus vaccine (RV1) in October, 2012. We aimed to investigate the impact and effectiveness of the RV1 vaccine in reducing diarrhoea-associated mortality in infants aged 10–51 weeks.

**Methods:**

In this population-based cohort study, we included infants born between Jan 1, 2012, and June 1, 2015, in Mchinji, Central Malawi and analysed data on those surviving 10 weeks. Individual vaccination status was extracted from caregiver-held records or report at home visits at 4 months and 1 year of age. Survival to 1 year was confirmed at home visit, or cause of death ascertained by verbal autopsy. We assessed impact (1 minus mortality rate ratio following vs before vaccine introduction) using Poisson regression. Among vaccine-eligible infants (born from Sept 17, 2012), we assessed effectiveness (1 minus hazard ratio) using Cox regression.

**Findings:**

Between Jan 1, 2012, and June 1, 2015, we recruited 48 672 livebirths in Mchinji, among whom 38 518 were vaccine-eligible and 37 570 survived to age 10 weeks. Two-dose versus zero-dose effectiveness analysis included 28 141 infants, of whom 101 had diarrhoea-associated death before 1 year of age. Diarrhoea-associated mortality declined by 31% (95% CI 1–52; p=0·04) after RV1 introduction. Effectiveness against diarrhoea-mortality was 34% (95% CI –28 to 66; p=0·22).

**Interpretation:**

RV1 was associated with substantial reduction in diarrhoea-associated deaths among infants in this rural sub-Saharan African setting. These data add considerable weight to evidence showing the impact of rotavirus vaccine programmes.

**Funding:**

Wellcome Trust and GlaxoSmithKline Biologicals.

## Introduction

Diarrhoea causes 17% of post-neonatal infant deaths globally.^1^ Despite impressive survival gains from improved sanitation and case management, rotavirus—the greatest contributor to this mortality—still caused 215 000 child deaths in 2013, 121 000 of these in Africa.^2^ Subsequently, with support from Gavi, the Vaccine Alliance, many African countries with the highest mortality burdens have introduced live attenuated rotavirus vaccines.^3^

Vaccine impact (ie, population reductions in disease burden following vaccine introduction) upon, and vaccine effectiveness (individual protection afforded by vaccination) against, rotavirus gastroenteritis hospitalisation have been shown^4^, ^5^, ^6^, ^7^ in high-income, middle-income, and low-income countries. Vaccine efficacy against laboratory-proven rotavirus in clinical trials is lower in low-income, high-mortality countries than in high-income, low-mortality countries.^8^ Therefore, to support widespread implementation, evidence of the impact of rotavirus vaccine on population-level mortality and real-world effectiveness on individual risk of death is crucially important. Vaccine impact on mortality has been shown^9^, ^10^, ^11^ through analysis of administrative datasets from middle-income countries in Central and South America. However, no direct mortality benefit of rotavirus vaccination has been documented at population level from a low-income, high-burden setting.

Malawi, a low-income country in sub-Saharan Africa, with year-round rotavirus transmission, has made sustained efforts to reduce child mortality and in 2015 had reached the Millennium Development Goal target of reducing child mortality by two-thirds from 1990 levels. In Malawi, health centres and community-based health surveillance assistants (HSAs; the community health-care workers and vaccinators in Malawi) routinely provide oral rehydration solution and zinc for diarrhoeal disease, which are widely available. 13-valent pneumococcal conjugate vaccine was introduced into Malawi's National Immunisation Programme with three doses given at 6, 10, and 14 weeks of age on Nov 12, 2011. Monovalent rotavirus vaccine (Rotarix, RV1) was introduced on Oct 29, 2012, at the WHO recommended schedule of 6 and 10 weeks, without catch-up. We have shown^6^, ^7^, ^12^, ^13^ RV1 efficacy (49%; 95% CI 19–68), effectiveness (64%; 95% CI 24–83), and impact (43%; 95% CI 18–61) on severe laboratory-confirmed rotavirus gastroenteritis in Malawian infants, and have shown that RV1 is highly cost-effective in this setting.

**Research in context**

**Evidence before this study**

Rotavirus vaccine (RV) has been introduced in many low-income countries with high mortality, supported by Gavi, but mortality impact or effectiveness estimates are absent from these settings. We searched PubMed using the terms “rotavirus vaccine [Title/Abstract]” AND “mortality [Title/Abstract]” OR “death [Title/Abstract]” NOT “review [Publication Type]” NOT “cost-effectiveness [Title]”. 185 citations arose from the search and NB-Z and CK reviewed the articles independently, excluding review articles and secondary publication of data. 13 studies, all from middle-income countries, were identified. A study in Botswana reported reductions in case fatality of infants in hospital of 48% and another one in Panama 45%, but neither reported on population mortality. All other studies (Bolivia, n=1; Brazil, n=5; Mexico, n=3; combined South American countries n=2) used time-series analyses of national administrative datasets to estimate mortality reductions following introduction of the rotavirus vaccine. These studies reported reductions in infant diarrhoeal-mortality of between 21% and 41%, with higher estimates noted within rotavirus season than outside the season. We did not identify any mortality impact data from low-income countries. To our knowledge, prospective, population-based studies investigating rotavirus vaccine impact on mortality have not been published from any country. In southern Malawi, RV1 introduction was associated with a 43% reduction in hospital admissions of infants with laboratory-confirmed rotavirus, with vaccine effectiveness of 64%, and it was highly cost-effective.

**Added value of this study**

To our knowledge, this large population-based birth cohort study is the first to report rotavirus vaccine-associated infant mortality reductions from a low-income country using the WHO recommended Expanded Programme on Immunisation schedule of 6 and 10 weeks, and shows an association between coverage achieved and mortality impact gained. Additionally, this study shows a possible added benefit on diarrhoeal mortality of vaccine introduction in the context of enhanced water, hygiene, and sanitation improvements.

**Implications of all the available evidence**

In addition to morbidity impact and high cost-effectiveness, countries with national or localised areas of high diarrhoeal mortality should consider introducing rotavirus vaccines for survival benefits. Vaccine implementation combined with improvement in water and sanitation might provide maximum impact.

We aimed to assess population-level impact and individual-level effectiveness of RV1 against diarrhoea-associated mortality using a large prospective population-based birth cohort in a rural population in Mchinji district, central Malawi (site 1). To support our estimate of RV1 programme impact, we also planned for a concurrent prespecified impact assessment in a smaller separate population in Chilumba, northern Malawi (site 2; appendix).^14^ We present results from the two sites in turn.

## Methods

Before study commencement, extensive community engagement and consultation activities were undertaken with Traditional Authorities, village chiefs, health committees, women's groups, district and environmental health officers, health-centre managers, and HSAs to ensure the study was welcome in communities and households.

Malawi's National Health Sciences Research Committee (#837) and the London School of Hygiene & Tropical Medicine (#6047) provided ethics approval.

### Site 1: data collection and management

To assess population-level impact and individual-level effectiveness, we did a large scale, prospective, population-based birth cohort study. Site 1 (in Mchinji district) population was 456 516 persons in the 2008 national census, with a crude birth rate of 32 births per 1000 population and postneonatal infant mortality rate of 28 deaths per 1000 livebirths in 2015.^15^, ^16^ The district is rural and borders Zambia and Mozambique. Its sparsely populated villages and agricultural estates are interspersed with semiurban trading centres. The economy is based on subsistence maize farming. Electricity is available in 3·3% of households.^16^ This district was the location of a previous cluster randomised trial,^17^ with strong community support for research. It had the requisite infrastructure to expand to district-wide mortality surveillance and allowed us to do this type of study.

We did a baseline district-wide census in March, 2012, to obtain household membership and create community-held household registers. To establish prospective household surveillance in 1832 census-enumerated villages within all 354 HSA clusters, we used a cadre of 1059 village-based key informants who were selected by village health committees. Key informants did continuous household surveillance and maintained updated paper-based household registers for about 100 households each, recording all pregnancies, birth outcomes, and deaths of children younger than 5 years, and of women of childbearing age. Key informants were supervised by and reported data monthly to 50 enumerators, who electronically scanned the updated registers. Enumerators did home visits to all liveborn infants at 4 and 12 months of age to record vaccination status and confirm survival. The system was supervised by eight monitoring and evaluation officers (MEOs). Deaths reported by informants were verified and specially trained MEOs determined cause of death by verbal autopsy captured electronically at the household, completed as culturally appropriate at least 2 weeks after death, by using the WHO 2012 verbal autopsy instrument (Open Data Kit software).^18^ We have published a detailed description of this surveillance system.^14^

Vaccine status was obtained from a scanned image of a vaccine record (health passport, which is held by the caregiver) issued by the government and caregiver report (completed during household visits by enumerators when infants were 4 and 12 months of age or by MEOs following death). Caregivers were asked directly about the receipt and date of each dose of every vaccine for which the child was age-eligible under the National Immunisation Programme. Vaccine status was cross checked against vaccination centre registers in a subset of records for quality assurance. Final vaccine status was determined per criteria outlined in the appendix. To compare reported versus recorded vaccine receipt, throughout recruitment mothers were interviewed by MEOs after infant vaccination at randomly allocated clinics. Additionally, throughout recruitment, enumerators collected sociodemographic data on maternal vitals, marital status, and educational level obtained, and data on house, water source, and sanitation quality. Quality controls were embedded in the database, which automatically triggered field checks in case of error or anomalous runs of data (eg, no births in a catchment for 3 months). MEOs met monthly to review data quality and timeliness and address field challenges.

Infants surviving to at least 10 weeks of age who were born between Jan 1, 2012, and Sept 16, 2012, constituted the prevaccination cohort. Those born between Sept 17, 2012 (ie, eligible for first dose of RV1 on the date of vaccine introduction), and June 1, 2015, constituted the vaccine-age eligible cohort. Impact analysis compared both cohorts, whereas analysis of individual survival for effectiveness was done in the vaccine-eligible cohort only. Livebirths were followed up when the child had reached 1 year of age or death, or were excluded if they migrated. 1-year follow-up concluded on June 1, 2016. Diarrhoea-associated death was defined as any deceased child whose caregiver reported non-bloody diarrhoea in the illness preceding death upon direct closed questioning at verbal autopsy.

### Site 1: statistical analysis

We derived vaccine programme impact as 1 minus diarrhoea-associated mortality rate ratio in the vaccine-eligible cohort versus prevaccination cohort using Poisson regression adjusted for sociodemographic covariates (table 1). The relative brevity of the prevaccine introduction period at site 1 precluded adjustment by year. We also restricted analysis to between January and June, months with known high rotavirus prevalence in Blantyre, Malawi.^19^ To examine the association between population vaccine coverage and mortality, we did a Poisson regression of the mortality rate against two-dose vaccine coverage (proportion of two-dose-eligible infants in the population who actually received both doses) over time and by HSA cluster.^17^ For HSA cluster analysis of mortality versus vaccine coverage, we also adjusted for cluster-specific means of household level sociodemographic covariates, but we had no data on communal assets such as state of roads or public infrastructure. When plotting mortality rates over time, we used locally weighted moving average smoothing (appendix).

**Table 1:** Vaccine-eligible cohort description and multivariable Cox proportional hazards survival analysis, site 1

	**Survived (n=28 718)**	**All-cause deaths (n=367)**	**Diarrhoea-associated deaths (n=108)**	Cox multivariable model, hazard ratio*(95% CI)	**p value**
**Rotavirus vaccine status**					
0 doses	1724 (6%)	65 (18%)	10 (9%)	1 (ref)	..
1 dose	563 (2%)	33 (9%)	7 (7%)	..	..
2 doses	26 086 (91%)	266 (72%)	91 (84%)	0·66 (0·34–1·28)	0·22
Missing	345 (1%)	3 (1%)	..	..	..
**Maternal marital status**					
Married	25 810 (90%)	283 (77%)	83 (77%)	1 (ref)	..
Single	1567 (5%)	39 (11%)	11 (10%)	1·91 (1·00–3·65)	0·05
Divorced or widowed	1287 (5%)	33 (9%)	9 (8%)	1·55 (0·74–3·27)	0·25
Died	20 (0·1%)	9 (2%)	5 (5%)	98·1 (39·5–243·6)	<0·001
Missing	34 (0·1%)	3 (1%)	..	..	..
**Maternal education**					
None	3173 (11%)	46 (13%)	13 (12%)	1 (ref)	..
Primary	21 963 (77%)	280 (76%)	82 (76%)	1·12 (0·59–2·11)	0·73
Secondary or tertiary	3543 (12%)	37 (10%)	13 (12%)	0·95 (0·40–2·27)	0·91
Missing	39 (0·1%)	4 (1%)	..	..	..
**Water source**					
Protected source	23 525 (82%)	283 (77%)	81 (75%)	1 (ref)	..
Open source	5167 (18%)	81 (22%)	27 (25%)	1·42 (0·90–2·24)	0·13
Missing	26 (0·1%)	3 (1%)	..	..	..
**Toilet facility**					
No facility	5186 (18%)	6 3 (17%)	20 (19%)	1 (ref)	..
Some facility	23 503 (82%)	301 (82%)	88 (81%)	1·30 (0·76–2·21)	0·34
Missing	29 (0·1%)	3 (1%)	..	..	..
**House quality**†					
Worst	21 922 (76%)	297 (81%)	86 (80%)	1 (ref)	..
Middle	4302 (15%)	41 (11%)	11 (10%)	0·90 (0·48–1·72)	0·76
Best	2464 (9%)	26 (7%)	11 (10%)	1·71 (0·84–3·46)	0·14
Missing	33 (0·1%)	3 (1%)	..	..	..
**Season of birth**					
Dry	15 229 (53%)	202 (55%)	63 (58%)	1 (ref)	..
Rainy	13 489 (47%)	165 (45%)	45 (42%)	0·89 (0·60–1·31)	0·55
**Mean (SD)**					
Mother's age‡	26·0 (6·6)	27·1 (7·3)	27·9 (7·9)	..	..
Household assets§	1·5 (1·2)	1·2 (1·2)	1·1 (1·2)	0·72 (0·59–0·87)	0·001

Data are n (%), unless otherwise specified.

* Hazard ratio of diarrhoea-associated death.

† House quality is a composite of the construction materials used to make the roof, walls, and floor.

‡ Mother's age is standardised to be the age at birth of the child.

§ Household assets include bicycle, radio, ox cart and mobile phone.

We calculated two-dose versus zero-dose effectiveness as 1 minus hazard ratio using Cox proportional hazards modelling of diarrhoea-associated death occurring at 10–51 completed weeks of life. Because children might die from causes other than diarrhoea, we also did competing risks–survival analysis. We used multivariable modelling to adjust for sociodemographic covariates using complete-case analysis (table 1). We have previously published^20^ the primary analysis plan and justification. In case of violation of the proportional hazards assumption and to better understand how effectiveness might be related to age, we did a fully parametric survival analysis using Royston-Parmar modelling.^21^ We examined whether cluster-level determinants influence individual level mortality hazard using random effects hierarchical models.

In our sentinel hospital in Blantyre, rotavirus prevalence in severe gastroenteritis was 35% overall and 51% in peak periods; we therefore presumed rotavirus prevalence of 45% in diarrhoea-associated deaths.^6^, ^22^ Given that our published effectiveness against rotavirus gastroenteritis in Malawian infants in hospital was 64%, we assumed that effectiveness against very severe rotavirus gastroenteritis (leading to death) would be higher at 70–80%. Applying a presumed 76% reduction to the 45% of deaths presumed attributable to rotavirus, gave an effectiveness of 34% against all-cause diarrhoea-associated death. Based on our established surveillance before RV1 introduction, we expected 1500 births per month and a postneonatal infant mortality rate of 18 per 1000 livebirths, of which six were diarrhoea-associated. We assumed 60% mean vaccine coverage over the recruitment period. Inflating for 12% loss to follow-up, we required 36 293 infants who survived to 10 weeks to obtain 80% power to detect effectiveness of more than 34%.

### Site 2: data collection and management

A demographic surveillance site (DSS) covering 35 000 individuals has operated in the remote lakeside region of Chilumba, northern Malawi since 2002.^23^ Crude birth rate was 30·8 per 1000 population in 2015, postneonatal infant mortality was 15 per 1000 livebirths, and electricity was available in 8·7% of households.^16^ This longstanding DSS provided robust data on historical mortality rates in infants before vaccine introduction from 2004 and was therefore considered useful for independent impact assessment. Individual survival analysis was precluded by the small total population. For this site, births, deaths, and migrations were reported monthly by village informants and validated in a rolling annual census (previously described).^23^ Verbal autopsies were done during home visits, as locally culturally appropriate, at least 2 weeks after death. Sociodemographic covariates and vaccine status were collected for age-eligible children at the time of census visit, with vaccination date transcribed from caregiver-held records (health passport) or caregiver reports. We used Poisson regression to test monthly diarrhoea-associated mortality rate among 10–51-week-old infants against vaccine coverage, adjusting for year to account for long-term trend.^24^ Unbeknown to us at planning phase, the Red Cross implemented rapid, widespread, and sustained water and sanitation interventions (WASH) across the DSS area alongside national vaccine introduction.^25^ Site 2 could therefore no longer serve its intended validation function, but afforded an unplanned opportunity to assess the combined impact of vaccination with WASH as a post-hoc analysis.

### Role of the funding source

Both study funders were provided the opportunity to review the study design. The funders had no role in data collection, analysis, or interpretation, or writing of the report. A preliminary version of this manuscript was reviewed by GlaxoSmithKline Biologicals for factual accuracy. All authors had full access to all study data. The authors are solely responsible for final content and interpretation, and share final responsibility for the decision to submit for publication.

## Results

**Figure 1:**
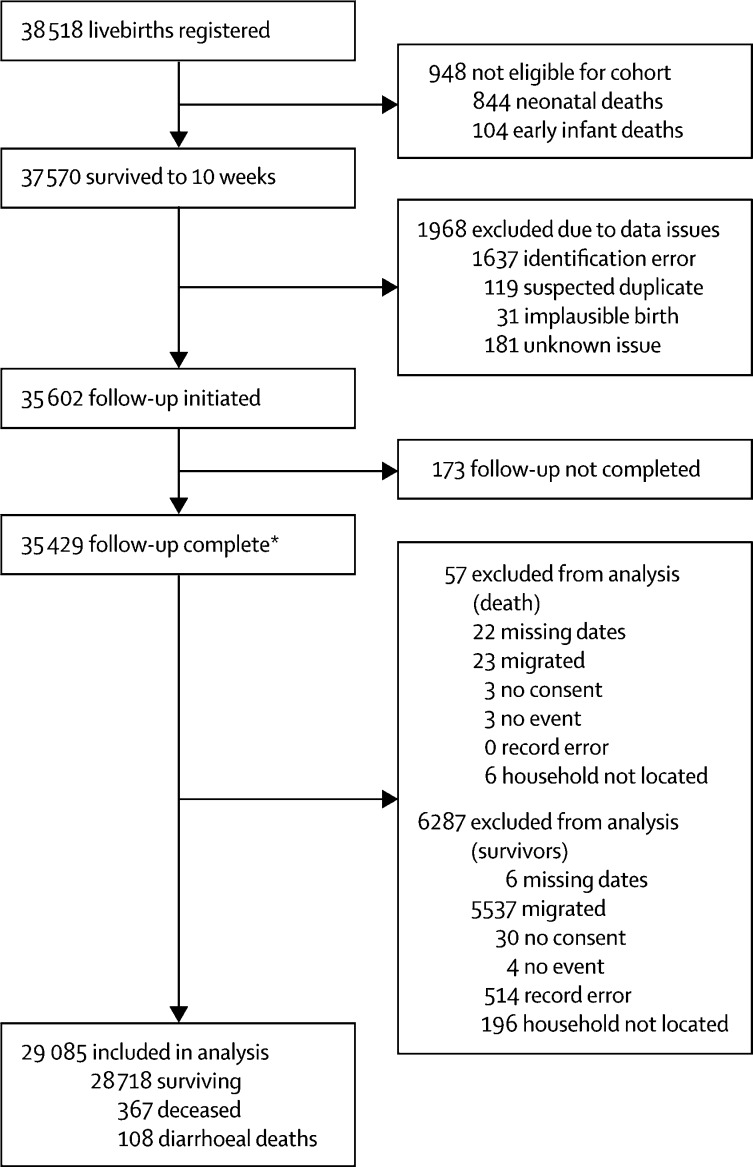
STROBE study profile of the participating vaccine-eligible cohort, Site 1 *Completion of follow-up means sufficient information was obtained by 1 year of age to determine whether the participant could be included in analysis or excluded for the reasons outlined in the figure.

For site 1, we registered 48 672 live births. Of these, the prevaccination cohort comprised 10 154 infants (born between Jan 1, 2012, and Sept 16, 2012), among whom 7818 infants survived to 10 weeks and were included in the analysis (appendix). The vaccine-eligible cohort included 38 518 infants (born between Sept 17, 2012, and June 1, 2015), among whom 37 570 infants survived to 10 weeks. 29 085 infants were included in the analysis, with 108 infants who died wtih diarrhoea before 1 year of age (figure 1). In the vaccine-eligible cohort, mean age at diarrhoea-associated death was 34 weeks, and 27 weeks for non-diarrhoea associated death (*t* test, p<0·001). Two-dose RV1 coverage was 90*·*6% overall, 90·8% in survivors, and 84*·*3% in deceased infants. Health passports were seen in 90% of infants overall, but ascertainment differed by survivorship—91% in survivors and 40% among the deceased. Sociodemographic factors were similar in survivors and deceased infants, except for maternal marital status or maternal death (table 1). Before RV1 introduction, compared with baseline assumptions (see Methods section), monthly births were 1112, postneonatal infant mortality rate was 18*·*8 per 1000 livebirths, diarrhoea-associated mortality was 5·6 per 1000 livebirths, and loss to follow-up was 18%. Post-hoc exploratory analysis found that infants lost to follow-up, compared with those who remained in the study, had younger (mean age: 25 *vs* 27 years) but more educated mothers (15% *vs* 12% secondary education) who were more likely to be unmarried (86% *vs* 89% married) and have slightly better housing quality (11% *vs* 9% best quality).

**Figure 2:**
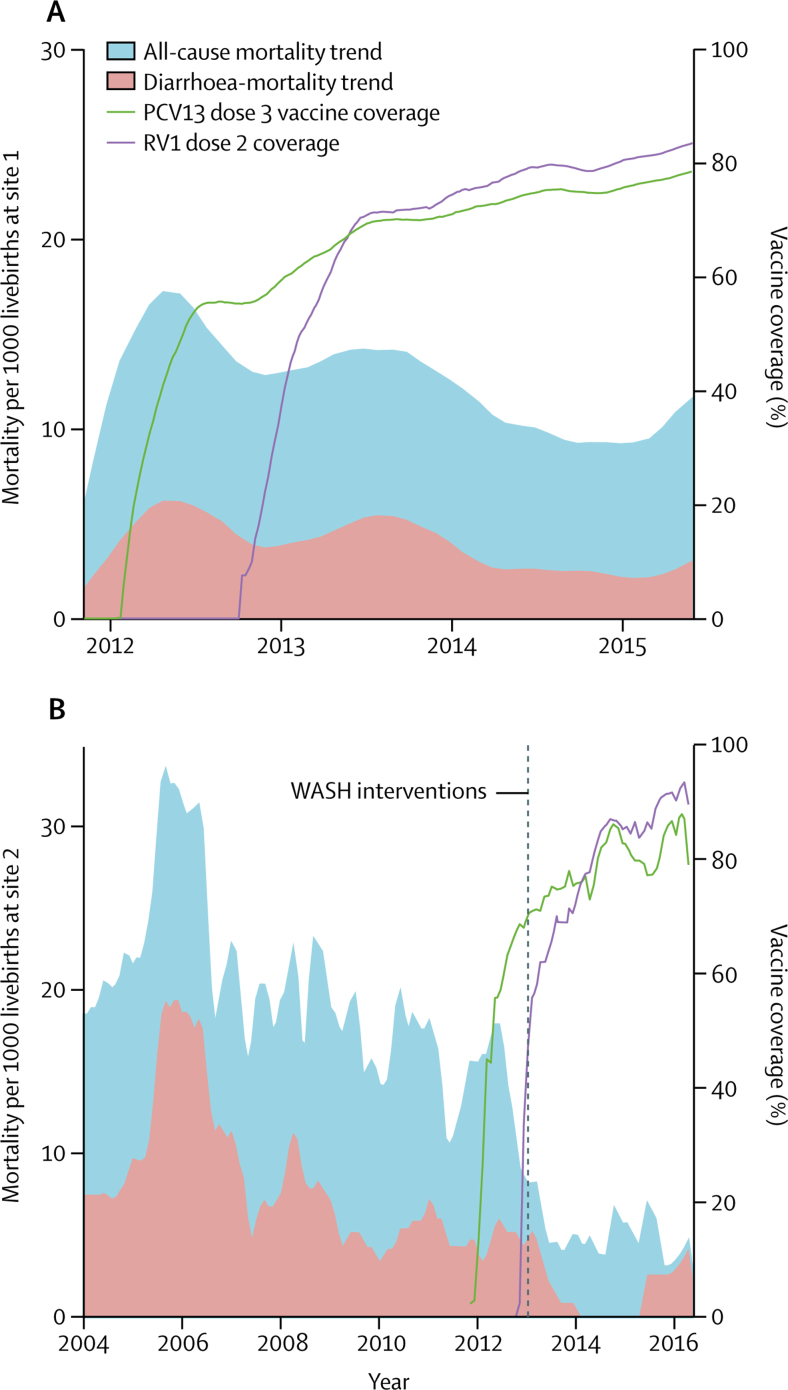
12-month weighted moving average smoothed trend* for all-cause and diarrhoea-associated mortality and dose 3 pneumococcal and dose 2 rotavirus vaccine coverage in 10–51-week old infants (A) Site 1; January, 2012, to June, 2015. (B) Site 2; January, 2004, to June, 2016. RV1=monovalent rotavirus vaccine. PCV=pneumococcal conjugate vaccine. WASH=water and sanitation. *See appendix.

Before vaccine introduction in the prevaccination cohort, 44 (mortality rate [MR] 5*·*6 per 1000 livebirths; figure 2A) of 7818 infants who survived to 10 weeks died with diarrhoea before 1 year of age. In the vaccine age-eligible cohort, 108 (MR 3*·*7 per 1000 live births) of 29 085 infants who survived to 10 weeks died of diarrhoea before 1 year of age. Unadjusted Poisson regression estimated vaccine impact on diarrhoea-associated mortality at 34% (95% CI 6–53; p=0*·*03; N=36 900) and sociodemographically adjusted Poisson regression estimated mortality at 31% (1–52; p=0*·*043; N=36 770). For equivalent January to June periods, assumed to represent peak rotavirus prevalence, in the postintroduction years 2013–15, the diarrhoea-associated mortality was 3*·*7 per 1000 in 2013 (impact 39% [95% CI 10–59]; p=0*·*013), 2*·*1 in 2014 (76% [58–86]; p<0*·*001), and 2*·*6 in 2015 (68% [47–81]; p=<0*·*001; table 2). All-cause mortality rate reduction post RV1 introduction was 25% (95% CI 8–39; p=0*·*008).

**Table 2:** Diarrhoea-associated death before and after RV1 introduction, site 1

	**Survived to 1 year**	**Diarrhoea-associated deaths**	**Diarrhoea-associated mortality rate per 1000**	**Vaccine coverage, % of eligible infants**	Vaccination impact*(95% CI); p value)
Prevaccination cohort	7690	44	5·6	NA	..
Vaccine eligible cohort	28 718	108	3·7	91%	31% (1–52); 0·043
January–June, 2012 (preRV1)	4232	28	6·6	NA	..
January–June, 2013	4339	16	3·7	89%	39% (10–59); 0·013
January–June, 2014	4180	9	2·1	94%	76% (58–86); <0·001
January–June, 2015	3830	10	2·6	95%	68% (47–81); <0·001

NA=not applicable. RV=rotavirus.

* 1 minus relative rate reduction in mortality following vaccine introduction compared with pre-introduction rate, using adjusted Poisson regression (adjusted for marital status, mother's education, quality of house, toilet and water source, and household assets).

In 354 HSA clusters of approximately 1300 persons each,^17^ mean postneonatal infant mortality was 12*·*3 per 1000 (SD 14·0; range 0–76*·*9) and diarrhoea-associated mortality was 3*·*6 per 1000 (SD 8·4; range 0–64*·*5). Two-dose vaccine coverage ranged from 63*·*6% to 100% across clusters; each percentage point increase in vaccine coverage was associated with a 1*·*6% (95% CI 0*·*8–2*·*5) lower diarrhoea-associated mortality rate (appendix). Adjusting for sociodemographic covariates, the reduction was 1*·*1% (95% CI 0*·*9–1*·*3)

**Figure 3:**
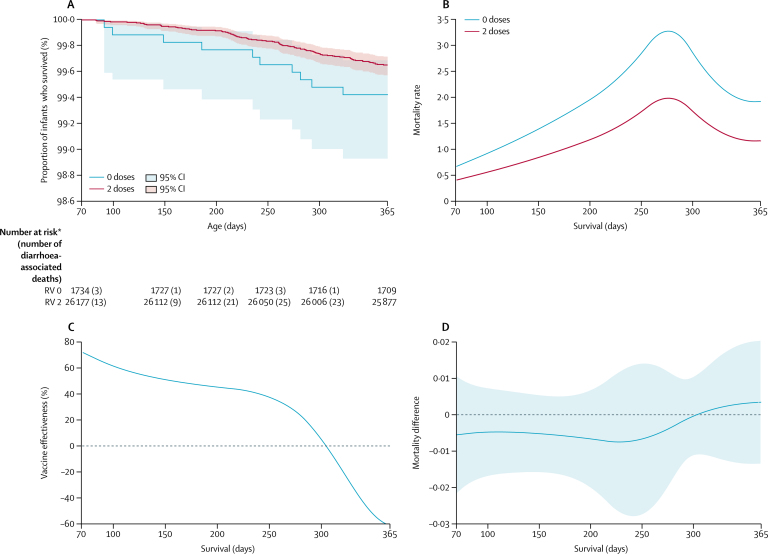
**Survival analysis of diarrhoea-associated death in the vaccine-eligible cohort, site 1** (A) Kaplan-Meier survival curve and 95% CIs, by vaccine receipt. (B) Fully parametric hazard rate over survival time, by vaccine receipt. (C) Vaccine effectiveness over survival time. (D) Hazard rate difference and 95% CIs (between vaccinated and unvaccinated infants) over survival time. RV=rotavirus vaccine. *Number at risk is the total number of surviving infants and infants who died with diarrhoea. 15 zero-dose and 209 two-dose recipients contributed to survival time until censoring for reasons other than death.

Of 26 352 fully RV1-vaccinated infants, 91 (0*·*4%) died with diarrhoea. In 1789 unvaccinated infants, ten (0*·*6%) died with diarrhoea (figure 3A). Unadjusted Cox modelling gave two-dose effectiveness against diarrhoea-associated mortality of 39% (95% CI –16 to 68) and for adjusted Cox modelling gave effectiveness of 34% (–28 to 66; table 1). Adjusting for HSA catchment area using a random effects hierarchical model gave an effectiveness of 36% (95% CI –24 to 67; likelihood ratio test p<0*·*001). Analysis of Schöenfeld residuals showed no evidence of violation of the proportional hazards assumption (p=0·23). Competing risks regression gave an effectiveness of 28% (95% CI –43 to 67). Effectiveness estimates derived from a Royston-Parmar model showed high effectiveness in early infancy, which declined after 6 months of age (figure 3C). Further sensitivity analyses and effectiveness against all-cause mortality are presented in the appendix.

For site 2, between Jan 1, 2004, and June 1, 2015, 15 394 livebirths were recorded. Of these 3531 were eligible for RV1, with 3433 infants surviving to 10 weeks. Follow-up was completed on June 1, 2016, for 3249 infants, with 3235 infants surviving to 1 year. Of the 14 deceased infants, three died with diarrhoea.

All-cause and diarrhoea-associated deaths have been declining since 2006, but were substantially lower since RV1 introduction and the Red Cross WASH interventions (figure 2B). Adjusting for year, to account for long-term trend, Poisson regression of raw data on monthly diarrhoea-associated mortality before and after these interventions gives a mortality-rate reduction of 46% (95% CI 26–60; p<0·001).

## Discussion

In this study, national introduction of RV1 was associated with a 31% reduction in diarrhoea-associated mortality in infants surviving to at least 10 weeks of age, and the degree of impact was strongly associated with vaccine coverage. Point estimate for individual protection from diarrhoea-associated mortality was 34%, though too few cases of diarrhoeal death occurred after introduction of vaccine to achieve sufficiently precise confidence bounds. In the context of published RV1 impact (43%) and effectiveness (64%) estimates against laboratory-confirmed rotavirus hospitalisation from Blantyre in southern Malawi, our estimates of impact (31%) and effectiveness (34%) against aetiologically non-specific diarrhoea-associated death have validity.^6^ The high effectiveness observed in months known to have high rotavirus prevalence (January to June) and the association between vaccine coverage and impact further attest to causal plausibility. These data from a low-income, high-burden setting therefore provide compelling evidence of RV1 impact on diarrhoea-associated infant mortality.

The estimates of mortality impact in site 1 are similar to those found in previous analyses of administrative datasets in middle-income countries.^9^, ^10^, ^11^, ^26^ For example, RV1 introduction in Mexico was associated with diarrhoeal-mortality rate reduction in infants of 41% and in Brazil of 21%.^9^, ^10^, ^26^ A study in Botswana, a sub-Saharan middle-income country, reported a 48% (95% CI 11–69) reduction in fatality among patients with gastroenteritis in hospital during the rotavirus season, and similar findings have been reported from a study in Panama; however, neither study measured population mortality.^27^, ^28^ The similar levels of protection found in our low-income sub-Saharan African setting is encouraging because children from this region account for more than half of global diarrhoea deaths, and with 31 African countries thus far introducing rotavirus vaccine, the absolute impact on mortality is likely to be substantial. 2, 3

The cohort design allowed us to estimate hazard and effectiveness by age—a metric that has been approximated in case-control studies.^29^ The observed hazard by age mimics the age at laboratory-confirmed rotavirus hospital admissions seen in our sentinel surveillance site in Blantyre (figure 2B). The apparent decline in effectiveness with age is unlikely to be due to individual immunological waning before 12 months, but it could be explained by changes in the force of infection through indirect effects.^13^ If rotavirus prevalence is declining (table 2), the hazard for unvaccinated infants declines so the measurable protection afforded by vaccine direct effects is thereby reduced. Survivorship bias might also contribute to lower effectiveness estimates in older infants since survivors who happen to receive vaccination late do not contribute their prevaccination survival time to the unvaccinated cohort, and survivors are implicitly more robust.

The greater individual level effectiveness against all-cause mortality than against diarrhoea-associated mortality (appendix) in site 1 is explained by confounding. Infants who did not receive RV1 had a greater likelihood of not receiving other Expanded Programme on Immunisation vaccinations, in particular pneumococcal vaccine that was introduced 10 months before RV1. Moreover, such children had greater association with other sociodemographic risk factors for mortality (appendix). Children from households with fewer assets had increased mortality hazard (table 1; appendix). We have previously published data^30^ from site 2 showing that sociodemographically vulnerable infants are at greater risk of both vaccine non-receipt and of death than those less vulnerable.

Our study has several limitations. First, on the one hand, vaccination population-impact evaluations are subject to temporal and secular biases, particularly for aetiologically non-specific endpoints. On the other hand, individual effectiveness estimates might be biased by access to vaccination or choice to vaccinate. We thus sought to determine both impact and effectiveness, and took account of sociodemographic confounding. However, successful vaccines with strong impact on disease incidence challenge sufficient accumulation of cases for individual-level analysis of adequate power, because deaths become rarer events. Thus, although the impact and effectiveness point estimates were similar, impact was such that effectiveness had wide confidence bounds. Second, although we inflated our sample size to account for anticipated loss to follow-up, it is possible that migrating children differed systematically from the rest of the population, thereby biasing vaccine effectiveness estimates. Single, wealthier, and more educated women were more mobile, but the differences, though nominally significant, were modest. The observed vaccine coverage and mortality rates in the non-migrating cohort aligned with our initial expectations. Third, retrospective updating of vaccine status might have been associated with bias toward higher apparent vaccine effectiveness.^31^ Coding vaccination date, as date of study ascertainment rather than the date vaccination actually occurred, might mitigate this bias, but this approach requires a high frequency of visits. Not only is this logistically challenging in a study of this magnitude, but might itself affect mortality outcome by increasing opportunity for illness recognition. Fourth, we went to great lengths to minimise underascertainment of both unvaccinated survivors and vaccinated infants who died, as previously described.^20^ Yet among deceased infants, health passports were often buried along with the child and unavailable for review. We could not change this cultural practice despite educational campaigns by radio and through community engagement. We actively sought vaccination clinic records to obtain vaccine status of deceased children, but finding the correct individual records of specific infants was challenging. We therefore assessed the quality of parental reporting through quality assurance activities. Our maternal exit interviews following vaccine clinic visits showed bidirectional misclassification of about 4% (data not shown). Restricting analysis to deceased infants whose records were available would itself have introduced bias. Fifth, cause of death misclassification can affect effectiveness estimation. Under-reporting of diarrhoea among vaccinated deceased infants will bias effectiveness and impact estimates away from the null. However, validation studies from Africa have shown high sensitivity for diarrhoea in effectiveness, and these are relatively robust to recall bias since parents remember the details of their child's final illness.^32^ Sixth, since date of vaccination was not always available, we could not analyse vaccination status as a time-varying covariate, which probably introduced a slight bias away from the null, since had we done so then the brief survival time between becoming eligible (we allowed 2 weeks for vaccination to be considered timely) and actually receiving vaccination would not have been included in vaccinated survival time. The fact that most vaccination was timely is therefore reassuring. Finally, other coadministered vaccines might also reduce diarrhoea-associated mortality, thus subtly increasing apparent RV1 effectiveness. Coadministration of other vaccines was almost universal, and we cannot account for this bias. In site 2, where we report a combined impact of RV1 introduction and a comprehensive WASH intervention, the magnitude of mortality reduction was 46%. Surveillance duration and therefore model adjustments differed across our two sites so the two results are not directly comparable. Given the unanticipated cointroduction of extensive improvements in sanitation at site 2, our result could have been biased away from the null because of other improvements in health care in this region, though in scoping with stakeholders we have not become aware of any other concurrent population interventions. Notwithstanding these caveats, the implication that concurrent interventions might have synergistic benefit is intriguing and warrants further programmatic evaluation.

Childhood diarrhoea-associated mortality in this rural African population has fallen during the past decade, in part because of improvements in sanitation and treatment interventions, including oral rehydration salts and zinc. Our large and comprehensive study shows for the first time, using empirically observed, population-based surveillance, that rotavirus vaccine is associated with a further reduction in diarrhoea deaths in a low-income, rural African population. These data add considerable weight to the WHO recommendation that all countries add rotavirus vaccine to existing public health interventions to further reduce diarrhoea deaths, particularly countries in south and southeast Asia and sub-Saharan Africa.^33^

**This online publication has been corrected. The corrected version first appeared at thelancet.com/lancetgh on August 21, 2018**
