# Correction to *Lancet Glob Health* 2018; 6: e1036–44

**DOI:** 10.1016/S2214-109X(18)30402-9

**Published:** 2018-08-22

*Bar-Zeev N, King C, Phiri T, et al. Impact of monovalent rotavirus vaccine on diarrhoea-associated post-neonatal infant mortality in rural communities in Malawi: a population-based birth cohort study.* Lancet Glob Health *2018; **6**: e1036–44*—The authors would like to change the license of this Open Access article to “CC BY 4.0 license”. This correction has been made as of August 21, 2018.

