# Supplementary material for: Impact of monovalent rotavirus vaccine on diarrhoea-associated post-neonatal infant mortality in rural communities in Malawi: a population-based birth cohort study
Source: Lancet Glob Health. 2018 Aug 10;6(9):e1036–44. doi: 10.1016/S2214-109X(18)30314-0 (PMC6088152; doi:10.1016/S2214-109X(18)30314-0)
Supplement: Supplementary appendix [file mmc1.pdf]

THE LANCET
Global Health

Supplementary appendix

This appendix formed part of the original submission and has been peer reviewed. We post it as supplied by the authors.

Supplement to: Bar-Zeev N, King C, Phiri T, et al. Impact of monovalent rotavirus vaccine on diarrhoea-associated post-neonatal infant mortality in rural communities in Malawi: a population-based birth cohort study. *Lancet Glob Health* 2018; **6:** e1036–44.

Appendix Materials

Appendix 1: Additional figures

Appendix Figure 1: Map of Malawi, study sites marked in red

Appendix Figure 2: Site 1 pre-vaccination cohort flow diagram per STROBE guidelines

Appendix Figure 3: Poisson model predicted diarrhoea-associated mortality vs vaccine coverage, Site 1

Appendix 2: Sensitivity analysis using different survival cut-offs and investigating random effects

Appendix 3: Socio-demographic status, Site 1

Appendix 4: Vaccine status construction

Appendix 5: Smoothing of mortality over time in Figure 2

Appendix 1: Additional figures

Appendix Figure 1: Map of Malawi, study sites marked in red

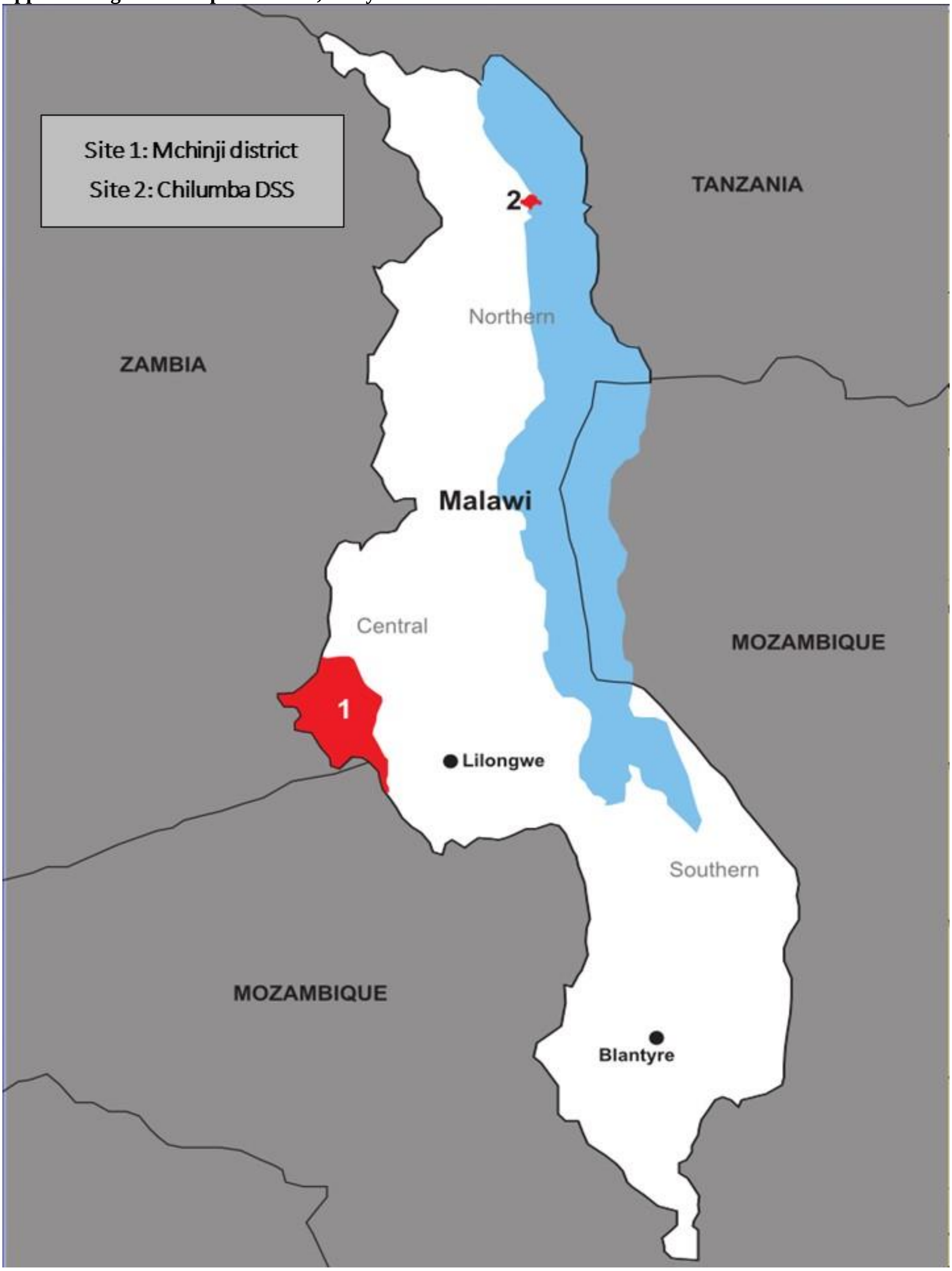

Appendix Figure 2: Site 1 pre-vaccination cohort flow diagram per STROBE guidelines

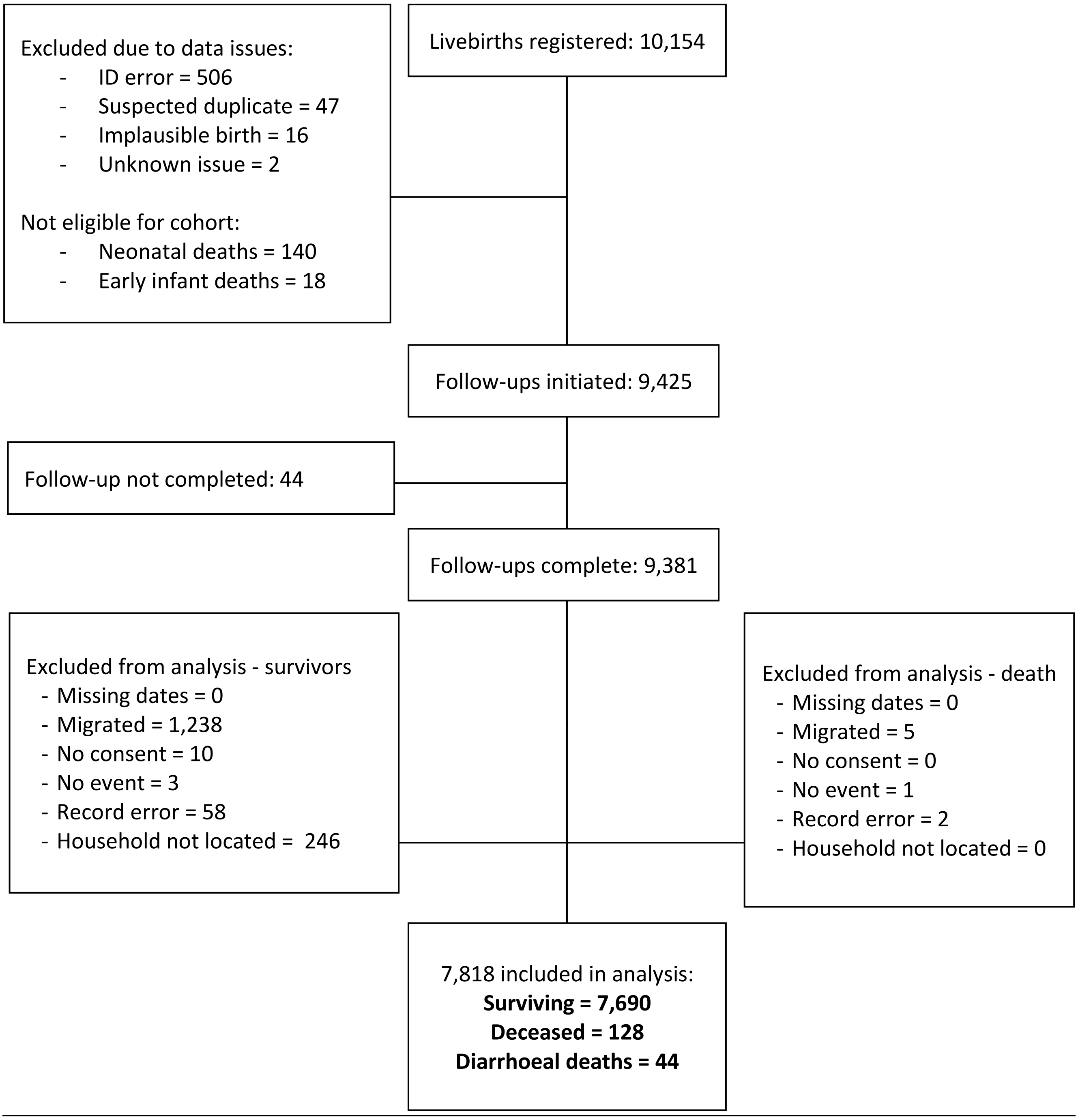

Appendix Figure 3: Poisson model predicted diarrhoea-associated mortality vs vaccine coverage, Site 1.

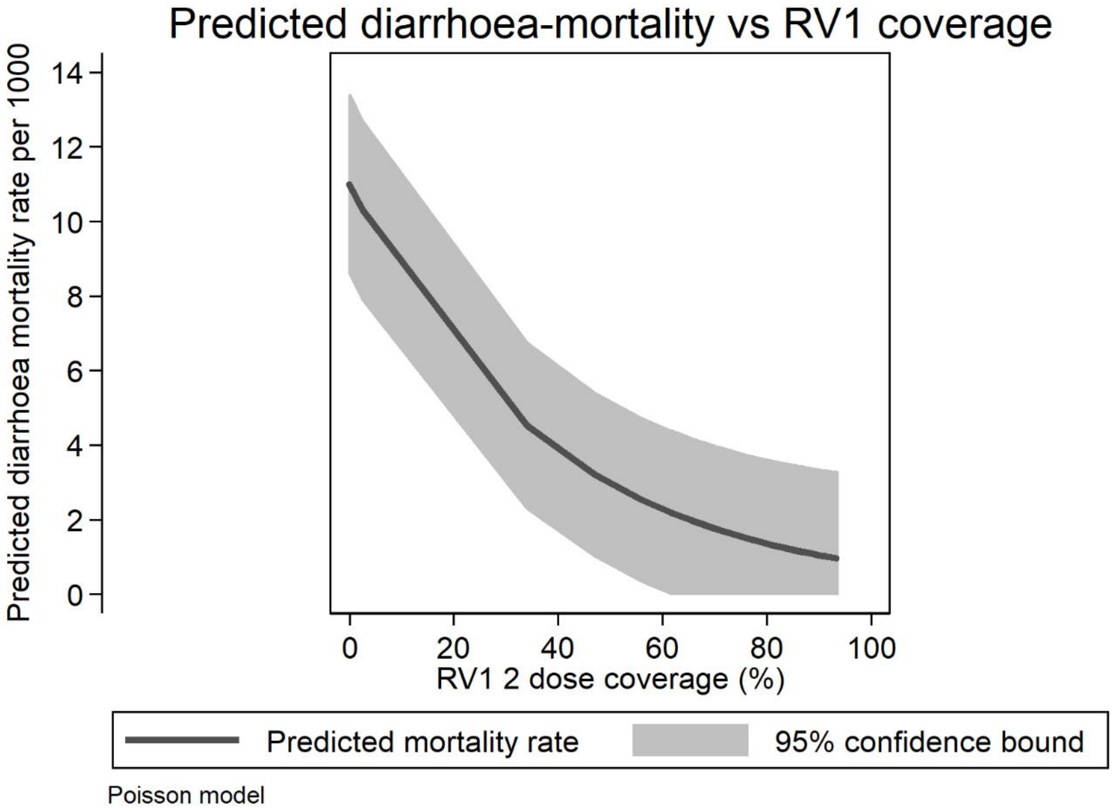

Appendix 2: Sensitivity survival analyses, Site 1

2.1 InterVA defined diarrhoea outcome (10wk survival)

Variable		Hazard Ratio	95% Confidence Interval		p-value
RV status	0 doses	1·00			
	2 doses	0·71	0·21	2·35	0·574
Mother's status	Married	1·00			
	Single	2·34	0·79	6·93	0·126
	Divorced/widow	1·85	0·54	6·34	0·330
	Deceased	66·60	8·93	469·80	<0·001
Mother's education	None	1·00			
	Primary	0·57	0·23	1·42	0·230
	Secondary/Tertiary	0·44	0·10	1·89	0·271
Water source	Protected source	1·00			
	Open source	1·03	0·42	2·53	0·942
Toilet facility	None	1·00			
	Some facility	0·94	0·40	2·24	0·890
House quality	Worst	1·00			
	Middle	0·88	0·26	3·00	0·842
	Best	2·62	0·83	8·29	0·101
Household asset index		0·69	0·48	0·99	0·041

Global test of proportional hazards: 0·2942

Infants eligible for inclusion in this sensitivity analysis: 27,912 survived, 31 died

2.2 Cohort inclusion at 6 week survival

Variable		Hazard Ratio	95% Confidence Interval		p-value
RV status	0 doses	1·00			
	2 doses	0·57	0·31	1·04	0·066
Mother's status	Married	1·00			
	Single	1·85	0·97	3·52	0·061
	Divorced/widow	1·45	0·69	3·06	0·323
	Deceased	82·90	33·40	205·77	<0·001
Mother's education	None	1·00			
	Primary	1·00	0·55	1·80	0·990
	Secondary/Tertiary	0·85	0·37	1·96	0·700
Water source	Protected source	1·00			
	Open source	1·42	0·91	2·22	0·122
Toilet facility	None	1·00			
	Some facility	1·29	0·77	2·17	0·333
House quality	Worst	1·00			
	Middle	0·93	0·50	1·72	0·818
	Best	1·65	0·82	3·33	0·161
Household asset index		0·73	0·61	0·88	0·001

Global test of proportional hazards: 0·447

Infants eligible for inclusion in this sensitivity analysis: Survived = 28,342, died = 105

2.3 Cohort inclusion at 26 week survival

Variable		Hazard Ratio	95% Confidence Interval		p-value
RV status	0 doses	1·00			
	2 doses	0·72	0·33	1·58	0·412
Mother's status	Married	1·00			
	Single	1·69	0·76	3·76	0·199
	Divorced/widow	1·92	0·86	4·31	0·111
	Deceased	136·81	54·62	342·69	<0·001
Mother's education	None	1·00			
	Primary	1·17	0·56	2·47	0·675
	Secondary/Tertiary	1·08	0·40	2·89	0·881
Water source	Protected source	1·00			
	Open source	1·42	0·84	2·39	0·188
Toilet facility	None	1·00			
	Some facility	1·25	0·68	2·30	0·471
House quality	Worst	1·00			
	Middle	0·93	0·46	1·91	0·853
	Best	1·44	0·63	3·34	0·389
Household asset index		0·77	0·62	0·96	0·020

Global test of proportional hazards: 0·665

Infants eligible for inclusion in this sensitivity analysis: Survived = 27,718, died = 77

2.4 Any dose of RV versus 0 doses (10 week cohort inclusion)

Variable		Hazard Ratio	95% Confidence Interval		p-value
RV status	0 doses	1·00			
	≥1 dose	0·62	0·32	1·20	0·156
Mother's status	Married	1·00			
	Single	1·87	0·95	3·68	0·071
	Divorced/widow	1·42	0·65	3·14	0·382
	Deceased	94·73	37·47	239·49	<0·001
Mother's education	None	1·00			
	Primary	1·43	0·70	2·89	0·324
	Secondary/Tertiary	1·48	0·60	3·66	0. ·393
Water source	Protected source	1·00			
	Open source	1·56	0·99	2·44	0·053
Toilet facility	None	1·00			
	Some facility	1·24	0·73	2·12	0·422
House quality	Worst	1·00			
	Middle	0·90	0·47	1·71	0·740
	Best	1·65	0·83	3·28	0·151
Household asset index		0·77	0·63	0·93	0·006

Global test of proportional hazards: 0·779

Infants eligible for inclusion in this sensitivity analysis: Survived = 28,012, died = 101

2.5 All-cause non-traumatic mortality (10 week cohort inclusion)

Variable		Hazard Ratio	95% Confidence Interval		p-value
RV status	0 doses	1·00			
	2 doses	0·29	0·22	0·38	<0·001
Mother's status	Married	1·00			
	Single	2·27	1·59	3·23	<0·001
	Divorced/widow	1·97	1·33	2·92	0·001
	Deceased	49·13	24·25	99·56	<0·001
Mother's education	None	1·00			
	Primary	1·02	0·73	1·45	0·89
	Secondary/Tertiary	0·75	0·45	1·24	0·26
Water source	Protected source	1·00			
	Open source	1·27	0·98	1·65	0·07
Toilet facility	None	1·00			
	Some facility	1·27	0·95	1·71	0·11
House quality	Worst	1·00			
	Middle	0·79	0·54	1·14	0·21
	Best	1·15	0·74	1·79	0·53
Household asset index		0·83	0·74	0·92	0·001

Global test of proportional hazards: 0·0002 (ie PH assumption is rejected)

Infants eligible for inclusion in this sensitivity analysis: Survived = 27,912, died = 317

Appendix 3: Socio-demographic status, Site 1.

3.1 Socio-demographic characteristics of children according to vaccination status

Variable		0 doses N	(%)	1 dose N	(%)	2 doses N	(%)
**TOTAL**		1,750		603		25,831	
Mother died		3	(0.2%)	0	(0%)	22	(0.1%)
Marital status:	Married	1,536	(88%)	541	(90%)	23,273	(90%)
	Single	95	(5%)	33	(5%)	1,406	(5%)
	Divorced/widow	114	(7%)	28	(5%)	1,133	(5%)
Education:	None	260	(15%)	103	(17%)	2,762	(11%)
	Primary	1,341	(77%)	447	(74%)	19,771	(77%)
	Secondary	148	(8%)	52	(9%)	3,285	(13%)
Water source	Open source	444	(25%)	129	(21%)	4,523	(18%)
	Protected source	1,304	(75%)	474	(79%)	21,308	(82%)
Toilet facility	No facility	394	(23%)	139	(23%)	4,561	(18%)
	Some facility	1,354	(77%)	464	(77%)	21,268	(82%)
House quality	Worst	1,399	(80%)	471	(78%)	19,675	(76%)
	Middle	234	(13%)	80	(13%)	3,906	(15%)
	Best	115	(7%)	52	(9%)	2,247	(9%)
		**Mean (SD)**		**Mean (SD)**		**Mean (SD)**	
Mother’s age		27·6	(6·76)	27·7	(7·30)	27·0	(6·58)
Household assets		1·33	(1·15)	1·36	(1·15)	1·55	(1·18)

3.2 Socio-demographic characteristics of entire cohort over time

	Year			
**Socio-demographic factor**	**2012**	**2013**	**2014**	**2015**
Any toilet facility	78·4%	79·9%	83·4%	85·1%
Household mobile phone ownership	38·5%	42·0%	44·6%	50·1%
No maternal education	13·6%	11·8%	10·4%	9·4%
Maternal primary education	74·2%	75·7%	77·3%	78·5%
Maternal secondary / tertiary education	11·8%	12·3%	12·3%	12·1%

Appendix 4: Vaccine status construction

There are three sources of vaccine status information available in Malawi:
- Health passports (government issued caregiver-held documents)
- Caregiver recall
- Under 1 government vaccine registers (filled by healthcare workers at the point of vaccination and stored in frontline health facilities)

Health passports were witnessed at home-visit interviews at 4 months and 1 year of age and at verbal autopsy interviews. Degree of reliability was then assigned to vaccine data source as outlined in the table, including relative merits of each source.

4.1 Vaccine data source reliability

Data Source	Strengths	Weaknesses	Reliability
Health passport	• Filled in at the point of vaccination<br>• Dates included<br>• Less than 5% mis-recording	• Differential availability according to survival status	High
Under 1 register	• Routine data, therefore should be available for all, irrespective of survival status	• Some registers are missing or of very poor quality<br>• Issues in tracing children through registers and across facilities<br>• Absence of record does not mean they are unvaccinated	Medium
Caregiver recall with known dates	• Dates included<br>• Generally some documented evidence provided e.g. twins health passport	• Uncommon	High
Caregiver recall of no vaccinations	• Generally anecdotal support which makes it believable	• Uncommon<br>• Relies on accurate recall	High
Caregiver recall	• Available for most children, regardless of survival status	• Recall bias and social-desirability bias (in both directions), so hard to adjust for the uncertainty<br>• Chance of interviewer bias	Low

The following hierarchical rules were applied to construct a binary variable indicating vaccine received or vaccine not received:

1. If at home visit interview or VA a vaccine is recorded as 'received' in the health passport, this information will be taken as correct.
2. If at home visit interview or VA a vaccine is recorded as 'not received' or 'missing', or where no health passport was seen:
   a. If available, the vaccine status from a health passport at any prior 4-month interview (if such occurred) will be used
   b. If vaccines have been recorded in the under 1 register with evidence of a date of vaccination, this vaccine status will be used
   c. If vaccine status is not determined by 1, 2a or 2b then caregiver report will be used.
3. In case of data conflict between 4-month visit, 1 year old visit, under-1 register or maternal report, information from the health passport will be prioritised, followed by under 1 register and then caregiver report.

Appendix 5: Smoothing of mortality over time in Figure 2

Figure 2 Panel A. shows locally weighted scatterplot smoothing function of all-cause and diarrhoea-associated mortality in 10-51 week old infants from Site 1 as implemented by Stata 13.1 on basis of Cleveland, W. S. 1979. Robust locally weighted regression and smoothing scatterplots. Journal of the American Statistical Association 74: 829-836.

Figure 2 Panel B. shows the 12-month locally weighted moving average smoothing function of all-cause and diarrhoea-associated mortality in 10-51 week old infants from Site 2. The longer duration of data prior to vaccine introduction allowed us to use 12-month weighting to demonstrate the trend over years. The function was defined as follows:

$$\hat{Y}_t = \frac{1}{24}(Y_{t-6} + Y_{t+6}) + \frac{1}{12}(Y_t + Y_{t-1} + Y_{t+1} + Y_{t-2} + Y_{t+2} + Y_{t-3} + Y_{t+3} + Y_{t-4} + Y_{t+4} + Y_{t-5} + Y_{t+5})$$

; where Y_t is the monthly observation at month t and $\hat{Y}_t$ is the locally-weighted estimate at month t.
